# Prevalence and clinical features of adult atopic dermatitis in tertiary hospitals of China

**DOI:** 10.1097/MD.0000000000006317

**Published:** 2017-03-24

**Authors:** Xin Wang, Xiao-Dong Shi, Lin-Feng Li, Ping Zhou, Yi-wei Shen, Qing-kun Song

**Affiliations:** a Department of Dermatology, Beijing Friendship Hospital, Capital Medical University; b Department of Dermatology, Beijing Shijitan Hospital, Capital Medical University; c Market Research Department, China Telecom Corporation Limited Beijing Research Institute, Beijing Future Science Park, Beijing, China.

**Keywords:** adult, atopic dermatitis, eczema, epidemiololgy

## Abstract

The prevalence of atopic dermatitis (AD) has increased substantially. Previous studies have focused mostly on pediatric patients, while epidemiological investigation on adult AD has been very limited.

The aim of this study was to determine the prevalence and clinical features of adult AD in outpatients with dermatitis and eczema in China mainland.

A multicenter cross-sectional study was conducted among outpatients with eczema or dermatitis from 39 tertiary hospitals of 15 provinces in China from July 1 to September 30, 2014.

Of 8758 patients, 407 were adult AD. Compared with adults with other types of dermatitis, the mean age (41.8 ± 14.3 vs 42.04 ± 15.38 years, *P* < 0.05) and onset age (35.2 ± 11.2 vs 39.2 ± 14.0 years, *P* < 0.001) of adult AD were younger, and mean disease duration was longer (5.3 ± 7.1 vs 2.8 ± 4.9 years, *P* < 0.001). About 53.3% adult AD involved 3 or more body locations, higher than adults with other types of dermatitis (34.4%, *P* < 0.001), but lower than those with pediatric and adolescent AD (73.8%, *P* < 0.001). History of asthma (19.2% vs 6.9%, *P* < 0.001) or allergic conjunctivitis (21.9% vs 14.9%, *P* < 0.05) was more common in adult AD than pediatric/adolescent AD. Suspected bacterial infection was more frequently in adult AD than adults with other types of dermatitis (24.3% vs 14.6%, *P* < 0.001) and pediatric/adolescent AD (24.3% vs 14.9%, *P* < 0.001). More severe itching was observed in 31.4% of adult AD, higher than that of adults with other types of dermatitis (15.4%, *P* < 0.001), whereas similar to that of pediatric/adolescent AD (28.7%, *P* > 0.05). The highest (8.7%) and lowest prevalence (3.7%) of adult AD were in 25°N to 30°N and 35°N to 40°N latitude region.

A substantial part of adult outpatients with eczema or dermatitis is adult AD. Middle age, more body location involvement, more suspected bacterial infection, and severe itching are the main clinical feathers of adult AD. Geographical environment and economic situation work in synergy to adult AD.

## 1 Introduction

Atopic dermatitis (AD) is a common dermatologic disease that significantly affects the quality of life. The prevalence of childhood AD ranged from 15% to 30%, while adult AD from 2% to 10% in industrialized countries.^[1]^ In China, the overall prevalence of AD in 2016 is reported to be 12.94% by clinical diagnosis of dermatologists, while based on UK diagnostic criteria, it is 4.76% in children aged 1 to 7 years.^[2]^ Very few studies have focused on adult AD, despite the fact that the occurrence of adult AD has obviously influenced socioeconomic conditions in both Asian and Western countries.^[3,4]^ According to a study in Taiwan from August 2007 to May 2008, 90 adult patients with AD (8%) were identified by dermatologists’ diagnosis among nursing staff in a Taiwanese medical center.^[5]^ Another study in USA involved a total of 3252 adults aged 18 to 85, 344 (6.2%) had AD.^[6]^ In China mainland, no epidemiological investigation of adult (≥18 years) AD has been conducted. Therefore, we performed a hospital-based, multicenter, cross-sectional epidemiologic survey of adult AD in Chinese outpatients with dermatitis and eczema, with a primary objective to determine the actual prevalence and clinical features of adult AD nowadays.

## 2 Methods

### 2.1 Study population

This study has been approved by Institutional Review Board (IRB) committee at each hospital involved in this study. Oral informed consent was acquired from each participate before enrollment. All procedures were conducted according to the guidelines approved by the ethics committee at each hospital. Demographic and clinical information was acquired from patients who were treated in the respective hospitals from July 1 to September 30, 2014. Patients were diagnosed with AD in 39 tertiary hospitals of 15 provinces and municipalities in mainland China, including Guangdong, Chongqing, Hunan, Jiangxi, Henan, Zhejiang, Shanghai, Hubei, Jiangsu, Anhui, Shanxi, Beijing, Tianjin, Shandong, and Liaoning Province, which generally represented most regions of China.

### 2.2 Diagnostic criteria of AD

All dermatologists involved in this study have abundant experience in clinical diagnosis and treatment for AD and have been trained in a standardized manner before the start of the project. First, each subject was inspected by a dermatologist independently. Then, a questionnaire survey was conducted by dermatologists after 10 to 15 minutes’ dermatological physical examination. All diagnostic criteria mentioned in Williams diagnostic criteria^[7]^ were integrated and recorded in detail; the parents or guardians had filled the consent forms for children patients. According to the questionnaire and physicians’ evaluation, a comprehensive diagnosis of AD was made on the basis of the gold standard “Williams diagnostic criteria” after investigation. And, we defined adult AD as AD patients with an onset age older than 18 years. Moreover, all patients with other types of dermatitis and eczema seen in the same period by the same investigators served as controls. Other types of dermatitis and eczema such as unclassified eczema, irritant contact dermatitis (ICD), widespread eczema, hand eczema, allergic contact dermatitis (ACD), neurodermatitis, seborrheicdermatitis, nummulareczema, asteatotic eczema, photo-contact dermatitis, autosensitization eczema, dyshidrotic eczema, and stasis dermatitis, were classified on the basis of International Classification of Diseases (ICD)-10^[8]^ and diagnosed accordingly on the basis of medical history and clinical features^[9]^. No laboratorial test was performed for dermatologists to make the diagnosis.

### 2.3 Data collection

All enrolled patients had completed a specific survey containing questionnaires regarding their general demographic characteristics, disease duration, severity of itching, lesion's distribution, type of skin lesion, and medical history. Itch was evaluated and divided into 4 levels: no itching; mild itching that interrupted neither daily activities nor sleep of the participant; moderate itching that interrupted daily activities but not affected sleep; and severe itching that affected both daily activities and sleep of the participant. History of allergic disease, dry skin, infantile eczema, and flexion dermatitis was also recorded. Allergic diseases included asthma, allergic rhinitis, allergic conjunctivitis, and AD. Secondary bacterial infection was clinically suspected if superficial pustules, prudent exudation, or yellow colored crust was detected.

### 2.4 Statistical analysis

All data was input into the Statistical Package for the Social Sciences software version 17.0 (IBM, NY) for statistical analysis. For continuous variables, the mean ± standard deviation (SD) was used according to the data distribution. The statistical methods included t-test χ2 test, and a correlation analysis. Differences in age and disease course between adult AD groups were evaluated by T-tests, and 95% confidence interval (95% CI) estimation with adjustment. Differences in gender, medical history, and suspected secondary bacterial infection between adult AD groups were analyzed by Chi-square tests. Itching grade between adult AD and other types of dermatitis was analyzed by Chi-square tests. The correlation between geographical location (20–25°N/25°01′–30°N/30°01′–35°N/35°01′–40°N/40°01′–45°N) and adult AD was analyzed by Spearman correlation test. Missing data were excluded from the analyses. All the analyses were two-tailed tests with the significant level of 0.05.

## 3 Results

### 3.1 Demographic characteristics

In total, 9393 patients were screened, of whom 636 cases (6.7%) were excluded due to incomplete information. Eight thousand seven hundred fifty-eight patients were recruited in the final analysis and 682 patients (7.8%) diagnosed with AD based on the Williams diagnostic criteria.^[7]^ Of them, 59.7% (407/682) of patients had adult AD, while 40.3% (275/682) had the disease onset before 18 years old, suggesting that a late-onset AD is very common. Overall, the prevalence of adult AD in outpatients with dermatitis and eczema was 4.6% (407/8758).

Although slightly more males than females (53.1% vs 46.9%) were affected, male predominance of adult AD was not seen in patients 18 to 28 years of age (male/female: 40.8% vs 59.2%). The age of adult AD was approximately normally distributed by P-P plot test, and most patients (55.0%) were middle aged (29–48 years). The mean age of adult AD was 41.8 ± 14.3 years (range = 71), which had no significant difference with that of adults with other types of dermatitis (42.04 ± 15.38 years, *P* = 0.715, 95% CI of difference = −1.70 to 1.17 years, *t* test).

### 3.2 Clinical characteristics

The comparison of clinical characteristics between adult AD and adults with other types of dermatitis is summarized in Table 1. The proportion of adult AD in AD was lower (407/682, 59.7%) than those of other types of dermatitis (6658/8076, 82.4%). The most often involved location of adult AD was fossa cubitalis (37.6%), followed by neck (34.4%) and knee (29.2%), which were more common than those of adults with other types of dermatitis (10.9%, 13.0%, and 9%, respectively, *P* < 0.0001). In addition, waist (26.3%) and upper limb (26.0%) were also frequently involved in adult AD. However, hand dermatitis was more common in adults with other types of dermatitis than adult AD (21.6% vs 17.2%, *P* = 0.036, Chi-square test). About 20.1% of adult AD had only 1 body location involved, while 53.3% had 3 or more body locations involved, which was higher than that of adults with other types of dermatitis (34.4%, *P* < 0.001), but lower than that of pediatric/adolescent AD (73.8%, *P* < 0.001, Chi-square test). Among 407 patients with adult AD, xerosis (224 patients, 55.0%), erythema (194 patients, 47.7%), and scratches (170 patients, 41.8%) were the 3 most common skin lesion types. Furthermore, vesicles (19.7% vs 13.8%, *P* = 0.004), pustule (15.7% vs 9.4%, *P* < 0.001), and erosion (16.5% vs 11.7%, *P* = 0.017) were more common in adult AD than adults with other types of dermatitis.

**Table 1:**
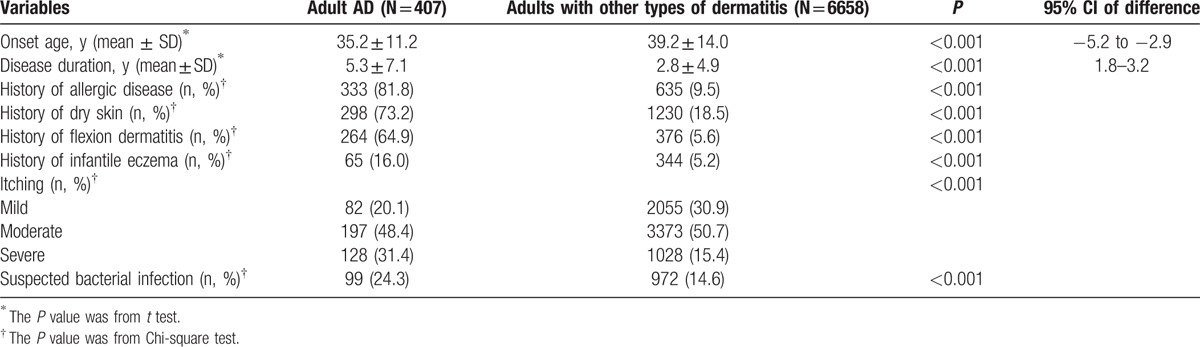
Clinical characteristics between adult AD and adults with other types of dermatitis.

The comparison of clinical characteristics between adult AD and pediatric/adolescent AD is summarized in Table 2. The waist (26.3% vs 17.5%) and foot (8.8% vs 4.0%) were more frequent sites involved in adult AD than pediatric/adolescent AD; however, other body locations were much less involved in adult AD. Face, eyelids, and ear were much less involved in adult AD than pediatric/adolescent AD (16.7% vs 51.3%, 7.1% vs 21.8%, and 9.6% vs 18.9%, respectively, *P* < 0.001, Chi-square test).

**Table 2:**
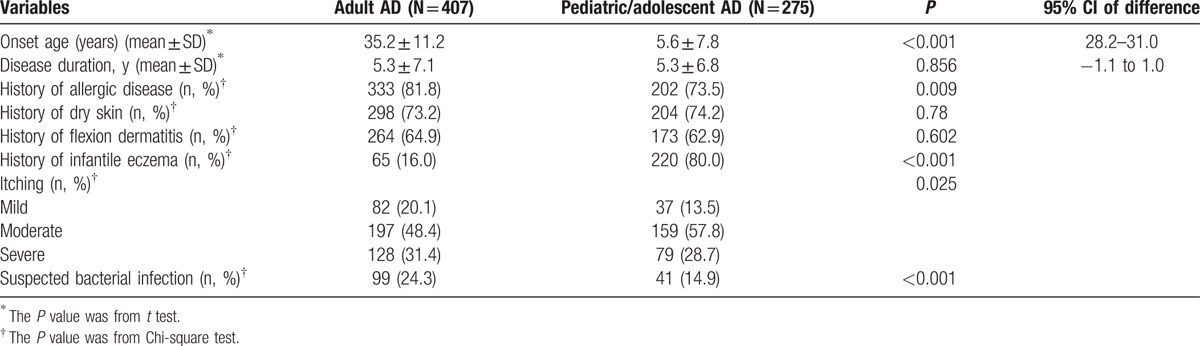
Clinical characteristics between adult AD and pediatric/adolescent AD.

More adult AD manifested as vesicles and nodules than pediatric/adolescent AD (19.7% vs 9.8%, and 13.8% vs 4.0%, respectively, *P* < 0.001). The most common skin lesion types of pediatric/adolescent AD were erythema (223/275, 81.1%), papula (201/275, 73.1%), and xerosis (167/275, 60.7%). History of allergic disease such as asthma (19.2% vs 6.9%, *P* < 0.001) and allergic conjunctivitis (21.9% vs 14.9%, *P* = 0.023) was more common in adult AD than pediatric/adolescent AD, but not for rhinitis (21.4% vs 21.8%, *P* = 0.89).

### 3.3 Geographical location

To analyze the relationship between geographical location and adult AD, 39 hospitals were divided into 5 groups based on latitude: 20°01′ to 25°N, Guangdong Province; 25°01′ to 30°N, Chongqing, Hunan, and Jiangxi Provinces; 30°01′ to 35°N, Henan, Zhejiang, Shanghai, Hubei, Jiangsu, Anhui, and Shanxi Provinces; 35°01′ to 40°N, Beijing, Tianjin, and Shandong province; and 40°01′ to 45°N, Liaoning Province. Although the average summertime temperature increased as latitude decreased, no trend of advancing prevalence of adult AD was observed with decreasing latitude.

The prevalence of adult AD and pediatric/adolescent AD in different latitude is summarized in Table 3. The highest prevalence (8.7%) of adult AD was in 25°N to 30°N latitude region and significantly higher relative to that of other regions (*P* < 0.001). The top 2 latitudes with a high prevalence of pediatric/adolescent AD were 35°N to 40 °N (4.4%) and 20°N to 25 °N (4.3%), with significantly higher prevalence than that of other latitudes (*P* < 0.001). By analyzing the ratio of AD in different latitudes, the top 2 latitudes with a high proportion of adult AD in all AD patients were 40°N to 45 °N (85.2%) and 25°N to 30 °N (79.7%), far above other latitudes (*P* < 0.001).

**Table 3:**
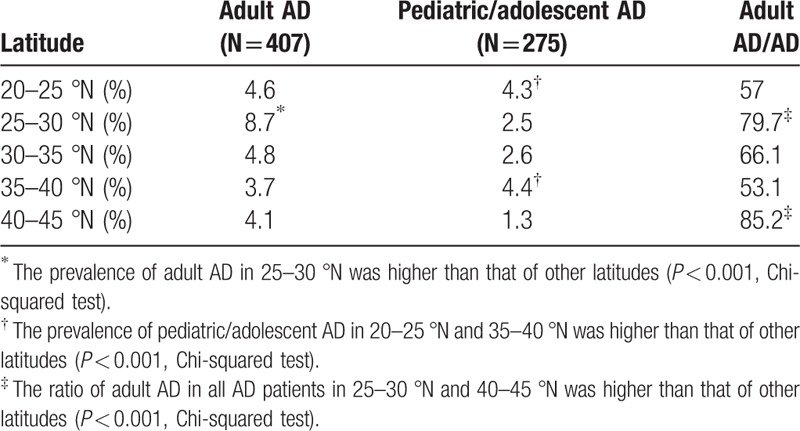
Prevalence of adult AD and pediatric/adolescent AD in different latitude.

## 4 Discussion

This study represents the first nationwide epidemiological survey of adult AD in outpatients in China mainland. The average age of adult AD was 40 years and the onset age was 35 years or so. As the middle-aged people are the major working force both in family and society, adult AD has become a great burden on public health. The longer disease course makes the situation even worse. That means adult AD has more lasting influence on the quality of life, indicating more attention should be paid on it.

The prevalence of adult AD in outpatients with dermatitis and eczema is 4.6% (407/8758), which is closer to that of children aged 1 to 7 years in China.^[2]^ It used to be that AD was an infantile disease, but in this investigation, the proportion of adults in AD is greater than children. Both the overall incidence of AD and the proportion of adults in AD are clearly on the rise in China, which attested late-onset AD is a common phenomenon. Both the awareness of adult AD by the public and the actual increase of the prevalence may contribute to this observation. With rapid economic development, the modes of production and living habits have changed dramatically, and environmental problems have been worsened. Although the occurrence of AD is associated with genetic factors,^[10,11]^ recent increase in incidence rate is more likely attributed to environmental factors,^[12–14]^ such as pollution and aeroallergens (e.g., pet hair, house dust mite, and pollen).^[15]^ In addition, changing lifestyles, such as frequent bathing, regular use of soap, and living in concrete jungles with air conditioning that dehumidifies the air, may accelerate stratum corneum (SC) barrier impairment.^[16]^

Guangdong Province, Shanghai, and Beijing are the best developed areas in China, located in 20°N to 25°N, 30°N to 35°N, 35°N to 40°N, respectively. In this investigation, we found that the prevalence of pediatric/adolescent AD was consistent with economic development, developed regions with higher pediatric/adolescent AD morbidity than that of other latitudes [35–40 °N (4.4%) and 20–25 °N (4.3%), *P* < 0.001, Table 3]. Because of overexposure to hostile environment or lifestyle urbanization in developed regions, people with strong genetic predispositions are more likely to develop AD in childhood. One interesting thing is the higher ratios of adult AD in all AD patients were located in economically undeveloped areas [40–45 °N (85.2%) and 25–30 °N (79.7%), Table 3], whether less exposure to risk factors may contribute the delayed onset age is worth to be studied. These phenomena prove indirectly the hypothesis that the influence and effect of external factors to susceptible populations need a cumulative process.

The climate generally has been considered as an influence factor of AD. A cohort study of 25 Irish children found that increased humidity and heat were associated with a higher incidence of disease atopic eczema flares.^[17]^ But in China, economic condition seems to give a more obvious effect on pediatric/adolescent AD than climate and geography. And in this study, no correlation was observed between advancing prevalence of adult AD and decreasing latitude. The highest (8.7%) prevalence of adult AD was in 25°N to 30°N latitude region, where it has relatively more humid air, higher temperature, and is underdeveloped. We assume that factors affecting prevalence of adult AD are more complicated and multi-faceted, and geographical environment and economic situation work in synergy to cause this result. Of course, this needs to be confirmed further.

Clinical characteristics of adult AD in China have not been fully studied. It has been well recognized that adult AD is characterized by a prominently red face and chronic lichenified eczema of the trunk.^[3,18]^ But red face is not very common in adult AD in our study (9.8%, 40/407). We notice that besides common body locations such as fossa cubitalis, neck, and popliteal fossa, waist and upper limb are also frequently involved. Perhaps because the waist in adults is more vulnerable to friction than children because of the use of belts, and upper limbs are more scratching relative to other parts. Whether or not the waist and upper limbs can serve as a diagnostic reference for adult AD requires a further investigation.

AD in early infancy is thought to contribute to the development of subsequent allergies known as atopic march, which is related to a damaged epidermal barrier.^[19]^ Atopy reflects a genetic predisposition for excessive IgE antibody production with 80% of children with AD generating serum concentrations of IgE typically 5-fold increased.^[20]^ AD is also associated with atopic diseases in other organs such as asthma and allergic rhinitis.^[21]^ More than half of children with moderate to severe AD develop allergic rhinitis and/or asthma, which are atopic disorders with significant morbidity and rare mortality.^[22]^ Current study demonstrates that history of asthma and allergic conjunctivitis, but not allergic rhinitis, is more common in adult AD than pediatric/adolescent AD. But in a study in Algeria, large differences were observed in the prevalence of asthma between age groups, this being highest in children aged under 16 and in the oldest group (>54 years).^[23]^ Although the mechanism underlying this difference is unclear, we have a bold vision that adult AD may have a different etiology from pediatric/adolescent AD, it is worth studying and analyzing.

Our study indicates that adult AD is especially prone to suspected secondary bacterial infection, perhaps because it mainly manifests as multiple scratch or erosion, leading to direct damage to the epidermal barrier, so that *Staphylococcus aureus* (*S. aureus*) can easily penetrate or invade the skin and feed off skin exudates.^[24]^ A major cause for SC barrier disruption in AD is attributed to loss-of-function mutations in *FLG* gene that encodes filaggrin, a structural protein in keratinocytes/corneocytes, thereby allowing outside-in penetration of foreign antigens and subsequent sensitization.^[25]^ Although a few recent studies have suggested that infant skin is more hydrated than adult skin, the skin microbiome of newborns resembles that of moist skin sites in adults.^[26]^ We found more adult AD manifested as vesicles, which perhaps one of the reasons for high prevalence of bacterial infection. In practice in China, the doctors do not grow bacterial cultures for each patient. This survey may have great implications in clinics, because it can optimize the choice of antibiotics.

Several limitations should be considered when interpreting our results: because participants were recruited from multiple tertiary referral hospitals located in provincial capitals or central cities, most patients visiting these hospitals were in a better financial status and medical insurance than the average. As a hospital-based study, a selective bias was inevitable due to a nonhomogeneous population and differential spatial distribution. Although the criteria of secondary bacterial infection were quite reliable,^[27]^ bacterial culture was required to confirm the infection. Some routine laboratory tests such as whole-blood count were not performed. We did not determine the final course of disease by long-term follow-up. All these factors may lead to an unavoidable bias.

To sum up, this study has provided an informative profile of adult AD in Chinese outpatients. Late-onset AD is a common subtype of AD, which has more lasting influence on the quality of life. Future studies are guaranteed to confirm its unique clinical characteristics.
